# Lactose in Human Breast Milk an Inducer of Innate Immunity with Implications for a Role in Intestinal Homeostasis

**DOI:** 10.1371/journal.pone.0053876

**Published:** 2013-01-10

**Authors:** Andreas Cederlund, Ylva Kai-Larsen, Gordana Printz, Hiroyuki Yoshio, Gunvor Alvelius, Hugo Lagercrantz, Roger Strömberg, Hans Jörnvall, Gudmundur H. Gudmundsson, Birgitta Agerberth

**Affiliations:** 1 Department of Medical Biochemistry and Biophysics, Karolinska Institutet, Stockholm, Sweden; 2 Department of Woman and Child Health, Karolinska University Hospital, Stockholm, Sweden; 3 Department of Neonatology, St. Marianna University School of Medicine, Miyamae-ku Sugao, Kawasaki, Japan; 4 Department of Biosciences and Nutrition, Karolinska Institutet, Stockholm, Sweden; 5 Institute of Biology, Biomedical Center, University of Iceland, Reykjavik, Iceland; Emory University School of Medicine, United States of America

## Abstract

Postpartum, infants have not yet established a fully functional adaptive immune system and are at risk of acquiring infections. Hence, newborns are dependent on the innate immune system with its antimicrobial peptides (AMPs) and proteins expressed at epithelial surfaces. Several factors in breast milk are known to confer immune protection, but which the decisive factors are and through which manner they work is unknown. Here, we isolated an AMP-inducing factor from human milk and identified it by electrospray mass spectrometry and NMR to be lactose. It induces the gene (*CAMP*) that encodes the only human cathelicidin LL-37 in colonic epithelial cells in a dose- and time-dependent manner. The induction was suppressed by two different p38 antagonists, indicating an effect via the p38-dependent pathway. Lactose also induced *CAMP* in the colonic epithelial cell line T84 and in THP-1 monocytes and macrophages. It further exhibited a synergistic effect with butyrate and phenylbutyrate on *CAMP* induction. Together, these results suggest an additional function of lactose in innate immunity by upregulating gastrointestinal AMPs that may lead to protection of the neonatal gut against pathogens and regulation of the microbiota of the infant.

## Introduction

The adaptive immune system of infants lacks antibodies specific for common pathogenic microbes and is deficient in differentiated immune cells [1]. This raises questions concerning the immune protection of newborns. Accumulating evidence suggests that neonates rely on innate immunity to combat infections [2]. Neonates must also establish their microbiota in order to generate a balance between immune defense and tolerance [3]. During this prolonged and sensitive period infants are susceptible to infections such as diarrheal diseases, one of the leading causes of child mortality [4].

Antimicrobial peptides (AMPs) and proteins are essential components in the defense against gastrointestinal infections, but also in shaping the microbiota [5]. Already at birth AMPs are present at several sites, such as the skin, *vernix caseosa*, and the respiratory and gastrointestinal tracts [6]–[8]. In addition, neonates obtain maternal immune support through factors in breast milk, like glycans [9], secretory IgA antibodies [10], lactoferrin [11] and AMPs, such as human β-defensins [12]. Moreover, milk nutrients contribute to the immune defense (reviewed in [13]). It is established that breast-fed infants harbor better immune defenses against several types of infections [14] and exhibit lower frequencies of inflammatory diseases than formula-fed infants [15], [16]. This implies that milk, apart from being a nutritional source, can augment the immune defense of infants. Studies on immunological factors of human breast milk have mainly focused on direct antimicrobial effects (reviewed in [10]) and less on the capacity to modulate the expression of infant immune genes.

Cathelicidins and defensins constitute major AMP families in mammals. In humans, the cathelicidin antimicrobial peptide (*CAMP*) gene encodes the sole human homolog, LL-37 [17], [18], an amphipatic, α-helical peptide derived from the inactive precursor hCAP-18 [19]. LL-37 is present in epithelial cells, monocytes, macrophages and neutrophils [17], [20], [21]. The expression is constitutive or can be induced by factors such as vitamin D and butyrate [22]–[24].

We have recently demonstrated that the level of LL-37 is higher in neonatal feces than in meconium [6], suggesting that milk factors can be important inducers of LL-37 expression. We here demonstrate that human breast milk induces the expression of *CAMP* in epithelial and monocytic cells and we identify lactose as the responsible intrinsic factor. We further show that the induction is mediated by p38 and JNK and that lactose together with butyrate or phenylbutyrate synergistically enhance *CAMP* gene expression. Thus, lactose has a hitherto unknown function in innate immunity.

## Materials and Methods

### Ethics Statement

The data were analyzed anonymously. Ethic permission is not applicable for these samples in accordance with Swedish law (2003∶460, §4). The human milk donors were informed both verbally and in writing that the samples were to be used for research purposes. Also, the parents of the infants whose fecal samples were analyzed were informed, both verbally and in writing, that the samples would be used for research. The parents of these neonates gave their consent verbally, and not in writing, in order to keep the samples anonymous. These consents are not documented. However, the parents anonymously filled out a form with information about the delivery type, breast feeding, and use of antibiotics.

### Milk Samples

Human breast milk, kindly donated from anonymous healthy mothers after verbal consent, was stored at −20°C until use. Commercially available formulas of seven different brands were prepared in accordance with manufacturer’s instructions. Milk and formula samples were extracted by the method of Folch [25]. Chloroform and methanol were added at a ratio of chloroform:methanol:milk 2∶1∶1. After shaking at room temperature for 30 min and centrifugation of 2300×g for 10 min, hydrophilic and hydrophobic fractions were separated, lyophilized and reconstituted in water and isopropanol, respectively.

### Chromatography of the Hydrophilic Fraction of Breast Milk

The hydrophilic fraction of breast milk was passed through a 10 kDa cut-off filter (Amicon Ultra, Millipore, Carrigtwohill, Ireland). The low-molecular weight fraction was fractionated on a cationic exchange column (140×16 mm, S-sepharose) (GE healthcare Life science, Uppsala, Sweden) using the ÄKTA HPLC system (GE Healthcare). The column was equilibrated in 0.2M acetic acid and a gradient of 0–5% buffer B was employed (0.2M acetic acid in 1.5M ammonium acetate) for 3.75 column volume (CV) at a flow rate of 1 ml/min and the column effluent was monitored at 230, 260 and 280 nm. Size exclusion chromatography was performed by injecting 400 µl of the active fraction from cationic exchange chromatography onto a Superdex peptide column (10×300 mm, GE Healthcare) in 100 mM ammonium acetate at a flow rate of 0.5 ml/min and column effluent was monitored at 230, 260 and 280 nm.

### Cell Cultures

The cell lines were obtained from American Type Culture Collection (Rockville, MD, USA): HT-29 (ATCC: HTB-38), Caco-2 (ATCC: HTB-37), T84 (ATCC: CCL-248) and THP-1 (ATCC: TIB-202). The human bronchial cell line VA-10, was a gift from Dr. Thorarinn Gudjonsson [26]. The HT-29 and THP-1 cells were propagated in RPMI 1640 (Sigma-Aldrich, St Louis, MO, USA) supplemented with 25 mM HEPES (Invitrogen, Carlsbad, CA, USA), 2 mM L-glutamine (Sigma) and 10% fetal calf serum (FCS) (Sigma). The Caco-2 cells were cultured in Dulbecco modified Eagles medium (DMEM, Invitrogen) supplemented with 20% FCS, 0.1 mM non-essential amino acids (Invitrogen). The VA-10 cells were cultured in bronchial epithelial cell basal medium (BEBM) (Lonza, Basel, Switzerland) [26]. T84 cells were cultured in a 1∶1 mixture of Ham’s F12 medium and DMEM supplemented with 2 mM L-glutamine and 5% FCS. For differentiation of THP-1 cells into macrophage-like cells these were incubated with 100 nM phorbol 12-myristate 13-acetate, PMA (Tocris Bioscience, Bristol, UK) for 48 h and only adherent cells were used for stimulations. Antibiotics (500units/ml penicillin and 500 µg/ml streptomycin) were added to all media. The cells were propagated at 37°C in 5% CO_2_, except for Caco-2, which were grown in 10% CO_2_. Before stimulation with breast milk fractions, formula or saccharides, cells were grown to ∼90% confluence and the medium was changed to serum-free. Cells were left untreated (unstimulated) or stimulated with different concentrations of breast milk fractions, or mono−/di-saccharides for 4, 24 or 48 h. After stimulation, the cell culture medium was centrifuged at 3500×*g* for 5 min; the supernatants were collected and stored at –20°C until further use. The cells were washed in PBS, treated with RNeasy mini kit lysis buffer (Qiagen) and kept at −80°C before cDNA preparation.

HT-29 cells were incubated with different inhibitors either alone or together with 60 g/l lactose for 48 h. The antagonists used were directed toward: p38 MAPK (10 µM SB203580 or 10 µM SB202190), c-Jun N-terminal kinase JNK (25 µM SP600125) and G-protein coupled receptors (1 µg/ml pertussis toxin). All antagonists were purchased from Tocris Bioscience. Furthermore, HT-29 cells were additionally induced with either 60 g/l lactose or 30 g/l galactose alone or in combination with 100 nM 1,25-dihydroxy-vitamin D_3_, 2 mM phenylbutyrate, or 2 mM sodium butyrate for 48 h.

### Immunological Detection of LL-37 in Cell Supernatants

**Figure 1:**
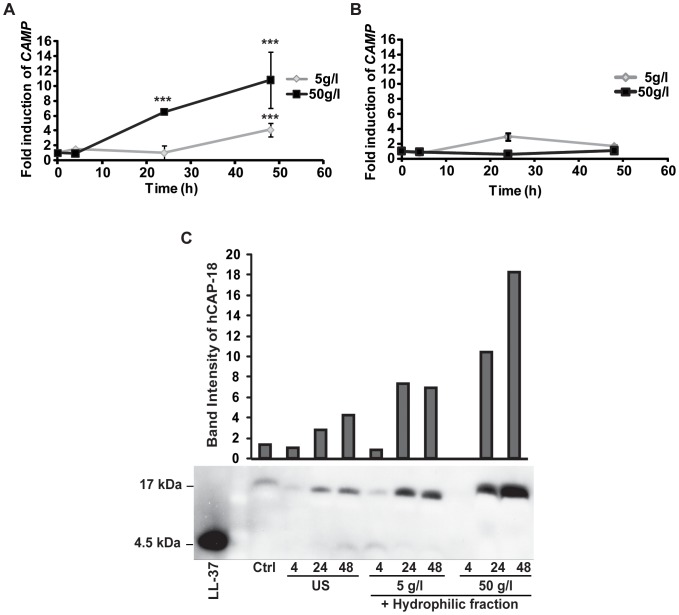
The hydrophilic fraction of breast milk induces the CAMP gene in HT-29 cells. (**A**) HT-29 cells were stimulated with 5 or 50 g/l hydrophilic fraction of breast milk for 4, 24 or 48 h. After 48 h a 10.8-fold increase of *CAMP* gene transcript was observed with 50 g/l. (**B**) Stimulation of HT-29 cells with the hydrophobic fraction of breast milk did not significantly induce *CAMP* gene expression. (**A–B**) Show means and standard deviations (SD) of at least three independent experiments performed in triplicates. (**C**) Western blot analysis showed induction of *CAMP* gene expression at the protein level. A low constitutive expression of the LL-37 proform hCAP-18 (17 kDa) was observed in the supernatants of unstimulated cells. hCAP-18 concentration increased after stimulation with both 5 and 50 g/l of the hydrophilic fraction with the highest increase after 48 h with 50 g/l. Control (ctrl) was 50 g/l of hydrophilic fraction mixed with cell medium from 48 hours unstimulated cells. Band intensities were calculated from one representative Western blot analysis using Image J and normalized to the band intensity of supernatants from cells propagated for 4 h in medium (US 4).

Protein enrichment of cell supernatants (2 ml) was performed as previously described [27]. Protein enriched eluates were lyophilized and stored at −20°C until further use. The lyophilized enriched proteins derived from cell supernatants were separated using sodium dodecyl sulfate polyacrylamide gel electrophoresis (SDS-PAGE) followed by Western blot analysis as described in [27]. Calculation of band intensity was performed using ImageJ (http://rsbweb.nih.gov/ij/). The intensity of each band corresponding to hCAP-18/LL-37 was quantified and calculated as ratio relative to the unstimulated control at 4 h (Fig. 1C, US 4).

### Spectrophotometric Measurements of Fecal Lactose and Galactose

Feces samples were collected from diapers of anonymous, healthy one week or one month old neonates after parents gave informed verbal consent. The fecal samples were extracted as described [6]. Lactose and galactose content was measured in accordance with manufacturer’s instructions using the Galactose and Lactose Assay Kit (Abnova, Taipei, Taiwan).

### Real-time Reverse Transcriptase PCR of CAMP Gene

Total RNA was extracted from cells using the RNeasy Mini Kit (Qiagen) according to the manufacturer’s instructions. cDNA from 1 µg RNA of each sample was synthesized by reversed transcriptase PCR (M-Moloney Murine Leukemia Virus, Invitrogen). Absolute quantification of cDNA was performed utilizing a CFX96 Real-Time system (Bio-Rad, Hercules, CA, USA). The *CAMP* gene expression was quantified using the fluorescent probe (5′-VIC-AAGGACGGGCTGGTG-TAMRA) and primers specific for *CAMP* cDNA were: forward 5′-TCACCAGAGGATTGTGACTTCAA; and reverse 5′-TCAGGGTCACTGTCCCCATAC. The 18S rRNA expression was investigated using the 18S rRNA housekeeping kit (Applied Biosystems), and presented as relative expression of *CAMP* gene transcripts in treated cells compared to untreated cells. No significant difference in 18S rRNA expression was observed of stimulated cells compared to control cells (data not shown). The experiments were performed in three independent experiments in triplicates.

### Mass Spectrometry

A QTOF 1 API instrument (Waters Corp. Milford, MA, USA) equipped with the standard Z-spray source was utilized. The samples were electrosprayed from a metal coated borosilicate glass capillary needle (Proxeon Biosystems A/S, Odense Denmark). The mass spectrometer was operated in positive ion mode at a resolution of 10,000 full width at half maximum height definition. The collision gas was argon with a pressure of 5.5×10^5^ mbar in the analysis penning read back. The running conditions were: capillary voltage of 1.2 kV, cone voltage and RF Lens 1 energy 90V and 38V, respectively. The source temperature was 80°C. Sample data was collected over 100–3000 *m/z* with a scan time of 1 scan/0.9 s and an interscan of 0.1 s for 5 min. The collision energy was set at 30 kV in MS/MS mode.

### NMR

A pooled sample of the most active fractions (40–42) from the size exclusion chromatography was lyophilized, dissolved in 0.5 ml D_2_O, lyophilized again and reconstituted in 0.5 ml D_2_O. This sample was then subjected to NMR-analysis using a Bruker Avance DRX 400 spectrometer. Proton-NMR analysis, ^13^C (APT), ^1^H-^1^H (COSY) correlated and ^1^H-^13^C (HMQC) correlated spectroscopy were performed. In addition, a commercial sample of α-lactose (5 mg, Sigma-Aldrich) was dissolved in 0.5 ml D_2_O, lyophilized, redissolved in D_2_O, left to equilibrate to the α/β isomer mixture at room temperature for 24 h, then subjected to Proton-NMR analysis and compared to the corresponding spectrum from the sample of the most active fractions.

### Statistical Analyses

Data are presented as means with standard deviations. Statistical differences between groups were assessed with Student two-tailed T-test (when samples were normally distributed) or the nonparametric Mann-Whitney test with a 95% confidence interval (when samples were not normally distributed). P-values below 0.05 were considered significant (*** p≤0.001, ** p≤0.005 * p≤0.05).

## Results

### CAMP Gene Expression is Induced by Breast Milk

**Figure 2:**
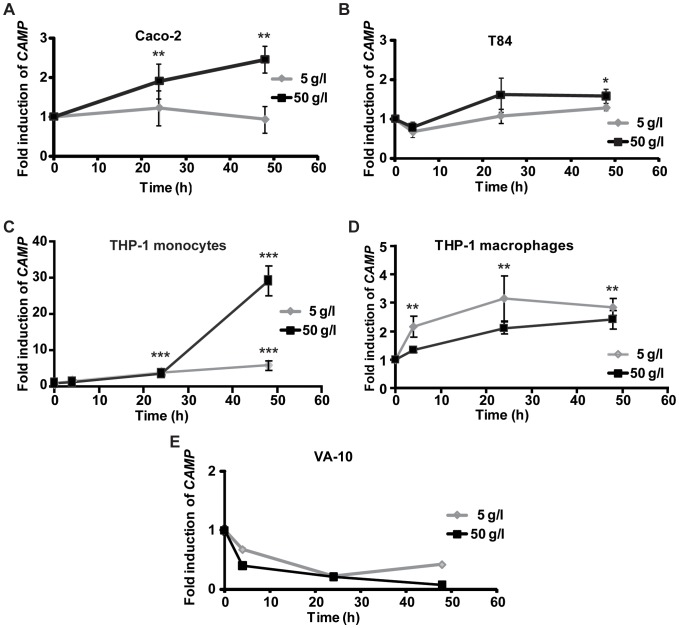
CAMP gene expression in cell lines after stimulation with the hydrophilic fraction of breast milk. (**A**) A significant induction of 2.5-fold of *CAMP* gene transcript was observed in Caco-2 cells after stimulation with 50 g/l at 24 and 48 h. (**B**) *CAMP* gene mRNA was 1.6-fold induced in T84 cells after both 24 and 48 h stimulation with 50 g/l. (**C**) A 29.2-fold induction of *CAMP* gene transcript was observed in THP-1 monocytes after 48 h stimulation with 50 g/l. (**D**) *CAMP* gene transcript was 3.2-fold induced after 24 h in THP-1 macrophages stimulated with 5 g/l. (**E**) No induction of *CAMP* gene was observed in VA-10 cells after stimulation with the hydrophilic fraction of breast milk (**A–E**) All graphs display means and SD of at least three independent experiments in triplicates.

We first separated human breast milk into a hydrophilic and a hydrophobic fraction using the Folch method [25]. The lyophilized fractions, reconstituted in water and isopropanol, respectively, were incubated at 5 or 50 g/l for 4, 24 or 48 h in HT-29 cells. Real-time PCR showed a significant induction (*** p≤0.001) of *CAMP* gene transcript upon stimulation with 50 g/l of the hydrophilic fraction (Fig. 1A), 6.5-fold after 24 h and 10.8-fold after 48 h. The hydrophilic fraction at 5 g/l (Fig. 1A) required 48 h for significant induction (*** p≤0.001) of *CAMP* gene transcript. Much lower or no induced expression of *CAMP* gene mRNA was observed with the hydrophobic fraction at both concentrations (Fig. 1B). Higher concentrations of the proform hCAP-18 were detected by Western blot analysis in supernatants from cells stimulated with the hydrophilic fraction than in unstimulated controls (US) (Fig. 1C). Only a low concentration of the mature peptide LL-37 was observed, indicating mRNA induction, translation and secretion of hCAP-18 with limited processing into LL-37. LL-37/hCAP-18 could not be detected in breast milk or in the hydrophilic fraction (data not shown), in contrast to previously reported results [28]. In addition, colonic epithelial cell lines Caco-2 and T84, THP-1 monocytes/macrophages, and the bronchial epithelial cell line VA-10 were also assayed for *CAMP* gene expression after stimulation with the hydrophilic fraction. Stimulation of Caco-2 and T84 cells with 50 g/l hydrophilic fraction resulted after 48 h in a significant increase in *CAMP* gene expression to 2.5 and 1.7-fold (** p≤0.005 and * p≤0.05), respectively (Fig. 2A and B). The same stimulation of THP-1 monocytes led to a 29.2-fold induction (*** p≤0.001) in *CAMP* gene transcript after 48 h (Fig. 2C). Furthermore, THP-1 macrophages stimulated with 5 g/l of hydrophilic fraction resulted in a 3.2-fold *CAMP* gene induction (** p≤0.005) after 24 h, while stimulation of THP-1 macrophages with 50 g/l hydrophilic fraction resulted in lower induction of *CAMP* gene (Fig. 2D). No induction of *CAMP* gene was observed in VA-10 cells at any dose or time assayed (Fig. 2E). These results indicate a cell-specific, and in THP-1 cells, differentiation-dependent response for induction of *CAMP* gene transcript by breast milk.

### Effects of Different Lactation Periods and Infant Formulas

**Figure 3:**
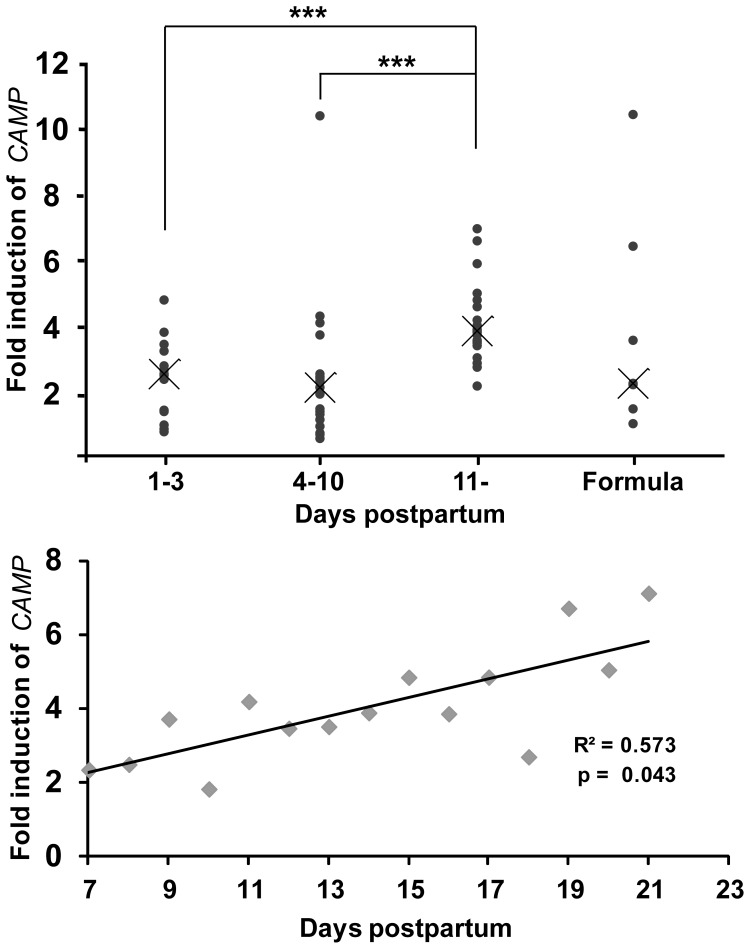
Relative inductions of CAMP gene transcript by colostrum, transitional milk, mature milk and infant formulas. (**A**) HT-29 cells were stimulated for 48 h with 50 g/l hydrophilic fractions of breast milk from different lactation periods. The median of the relative induction of *CAMP* gene transcript was 2.5-fold for colostrum (1–3 days postpartum, pp), 2.1-fold for transitional milk (4–10 days pp) and 3.9-fold for mature milk (11- days pp) samples. The median of 50 g/l hydrophilic fraction of infant formulas was 2.2, but with a high variability in *CAMP* gene induction. (**B**) HT-29 cells were stimulated with the hydrophilic fraction of breast milk collected from one mother from day 7 to 19 pp. A positive linear correlation was observed between *CAMP* gene transcript induction and time pp (R^2^ = 0.5728, p-value 0.043). (**A and B**) Each sample is performed in triplicates.

Hydrophilic fractions of breast milk from several donors of different lactation periods were used for stimulation of HT-29 cells at 50 g/l for 48 h, and the relative expression of *CAMP* gene transcript was compared. The median for colostrum (0–3 days postpartum, pp) and transitional milk (4–10 days pp), 2.5- and 2.1-fold, respectively, was significantly lower than the median for mature milk (11 days or later pp), which was 3.9-fold (Fig. 3A). Similarly, breast milk samples from one mother at different lactation periods were investigated for *CAMP* gene expression and showed a positive correlation (R^2^ = 0.573, p = 0.043) between induction and time pp (Fig. 3B). Thus, these experiments indicate that the inducing capacity of breast milk on *CAMP* gene expression increases with time postpartum. Seven brands of infant formula were also investigated for induction of *CAMP* gene mRNA and showed a large variation with a total median of 2.2-fold (Fig. 3A). No correlation between formula lactose concentration and *CAMP* gene induction was found.

### Isolation and Identification of Lactose as the CAMP Gene Inducing Compound

**Figure 4:**
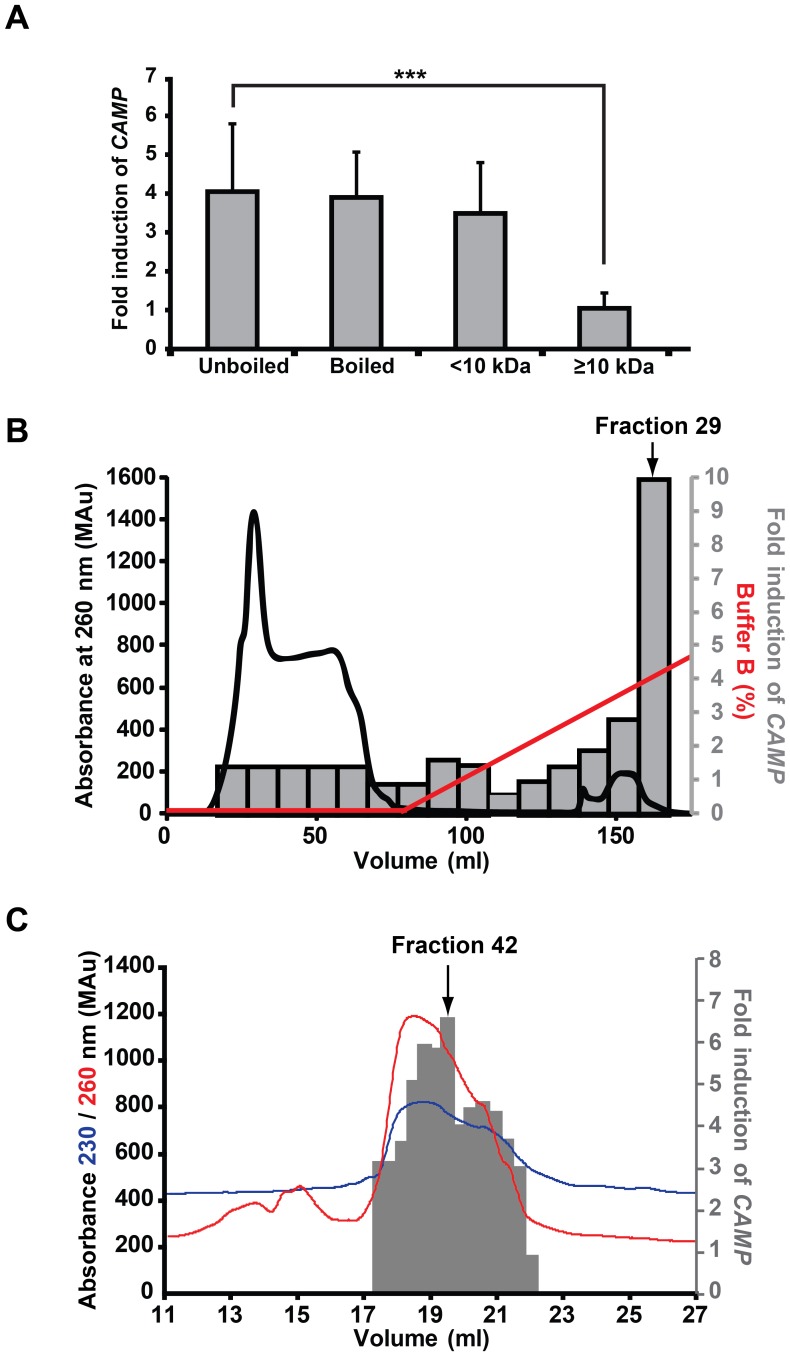
Isolation of the CAMP gene inducing component. (**A**) Heat treatment (100°C) of the hydrophilic fraction of breast milk for 30 min did not affect *CAMP* gene inducing capacity in HT-29 cells after 48 h of stimulation. Breast milk components were separated into high and low molecular weight components (more or less than 10 kDa) and the *CAMP* gene inducing capacity of breast milk was retained only in the low molecular weight fraction (≤10 kDa). Displays the mean and SD of at least three independent experiments in triplicate. (**B**) The low molecular fraction was subjected to cationic exchange and the chromatographic fractions were used for stimulation of HT-29 cells for 48 h. Material in fraction 29, eluted at 4% buffer B, resulted in a 10-fold induction of *CAMP* gene transcript. (**C**) Material in fraction 29, from (**B**), was separated by size exclusion chromatography and obtained fractions were assayed for *CAMP* gene transcript in HT-29 cells after 48 h of stimulation. A 6.5-fold induction of *CAMP* gene was observed by stimulation with material from fraction 42. (**B–C**) The X-axes denote the elution volume. The grey bars representing the activity are shown as a mean of the fold induction performed in triplicate.

The thermostability of the *CAMP* gene inducing components was assessed by heating the hydrophilic fraction to 100°C for 30 min, resulting in no reduction of its *CAMP* gene inducing capacity in HT-29 cells (Fig. 4A). The hydrophilic fraction was sub-fractionated into high (≥10 kDa) and low (<10 kDa) molecular weight fractions by ultrafiltration. Only the latter fraction could induce *CAMP* gene transcript (Fig. 4A). The low molecular weight fraction was further subjected to cationic exchange chromatography. Obtained fractions were screened for *CAMP* gene inducing capacity in HT-29 cells and fraction 29, eluting at ∼4% buffer B could induce *CAMP* gene transcript 10-fold (Fig. 4B, grey bars). No protein was detected in this material in a silver stained SDS-PAGE gel (data not shown). The material in fraction 29 was subjected to size-exclusion chromatography and stimulation of HT-29 cells with material from fraction 42 induced *CAMP* gene mRNA 6.5-fold (Fig. 4C, grey bars). Combined, these results indicate that the *CAMP* gene inducing component is non-proteinaceous, weakly cationic and of low molecular weight (190–1200 Da).

**Figure 5:**
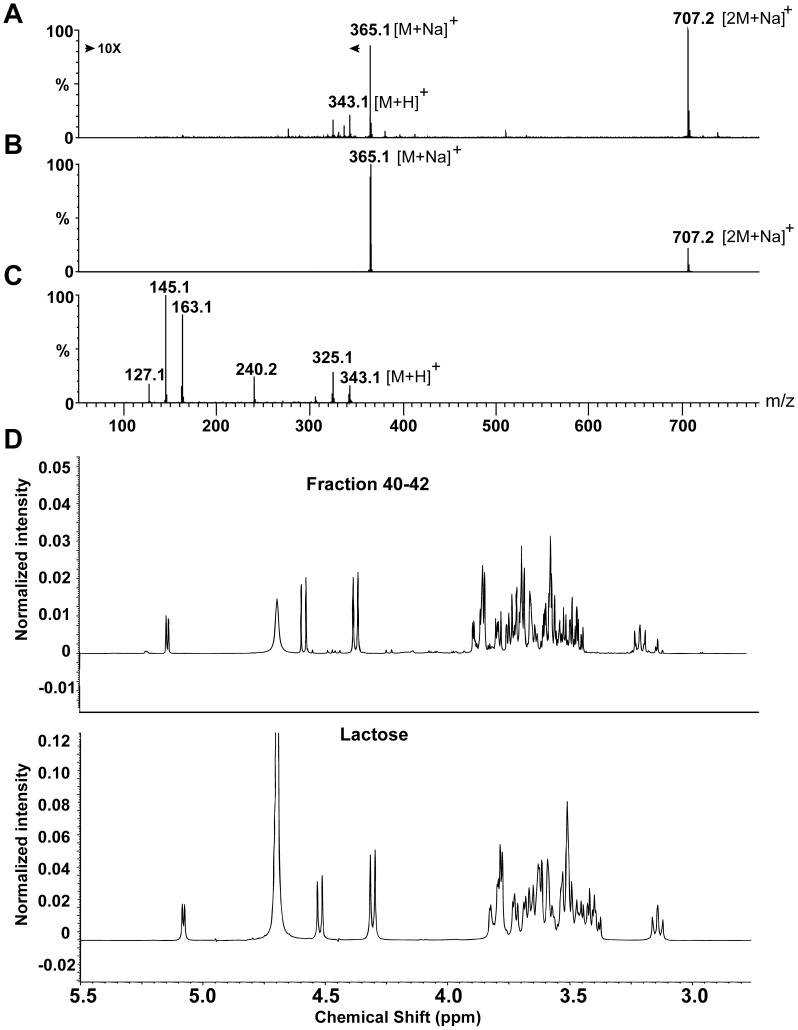
Characterization of the CAMP gene inducing component. (**A**) The intact molecular mass of the most abundant components in fraction 42 was 365.1 and 707.2 corresponding to a sodiated monomer of a disaccharide [342.1+ Na]^+^ and a sodiated dimer of a disaccharide [2×342.1+ Na]^+^, respectively. The mass of 343.1 corresponded to a protonated disaccharide [342.1+H]^+^. (**B**) Fragmentation of the 707.2 component resulted in a peak of 365.1, supporting the presence of disaccharides *i.e.* [342+Na]^+^. (**C**) Fragmentation of the 343.1 peak resulted in two peaks of 325.1 and 163.1, corresponding to a disaccharide and a monosaccharide with the elimination of water, respectively. (**D**) Top: ^1^H-NMR spectrum in D_2_O (at 20°C) of the pooled fractions 40–42. Bottom: ^1^H-NMR spectrum in D_2_O (at 20°C), of the equilibrated α/β mixture of lactose. These results establish lactose as the major component in the active fractions.

Material from the active fraction was subjected to electrospray ionization mass spectrometry (ESI-MS) analysis. The mass spectrum (Fig. 5A) shows the intact molecular weights of two major peaks, corresponding to the molecular weights of 365 and 707 Da. After subtracting the mass of a sodium adduct, giving 342 and 684 Da, masses proximate to those of a monomer and dimer of a disaccharide, respectively. In collision-induced dissociation (CID) experiments, fragmentation of the 707 Da component resulted in a fragment of 365 Da, further supporting the presence of disaccharides (Fig. 5B). Since sodiated components are difficult to fragment, the 342 Da component, and not the sodiated 365 Da component, was fragmented and resulted in several peaks corresponding to fragments of a protonated disaccharide. Fragments of 325 and 163 Da match the molecular weight of a disaccharide and a monosaccharide with the loss of one water molecule, respectively (Fig. 5C).

Material from the active fractions 40–42 was pooled, lyophilized, reconstituted in deuterated water and subjected to nuclear magnetic resonance (NMR) analysis. The major component was identified as a mixture of α,β-lactose with only minute traces of other compounds. The interpretation was further corroborated by comparison of ^1^H-NMR spectra of pure lactose and the pooled active fractions, which then unambiguously confirmed that the identity of the component was a mixture of α- and β-lactose (Fig. 5D).

### Lactose Induction is Dose- and Time-dependent

**Figure 6:**
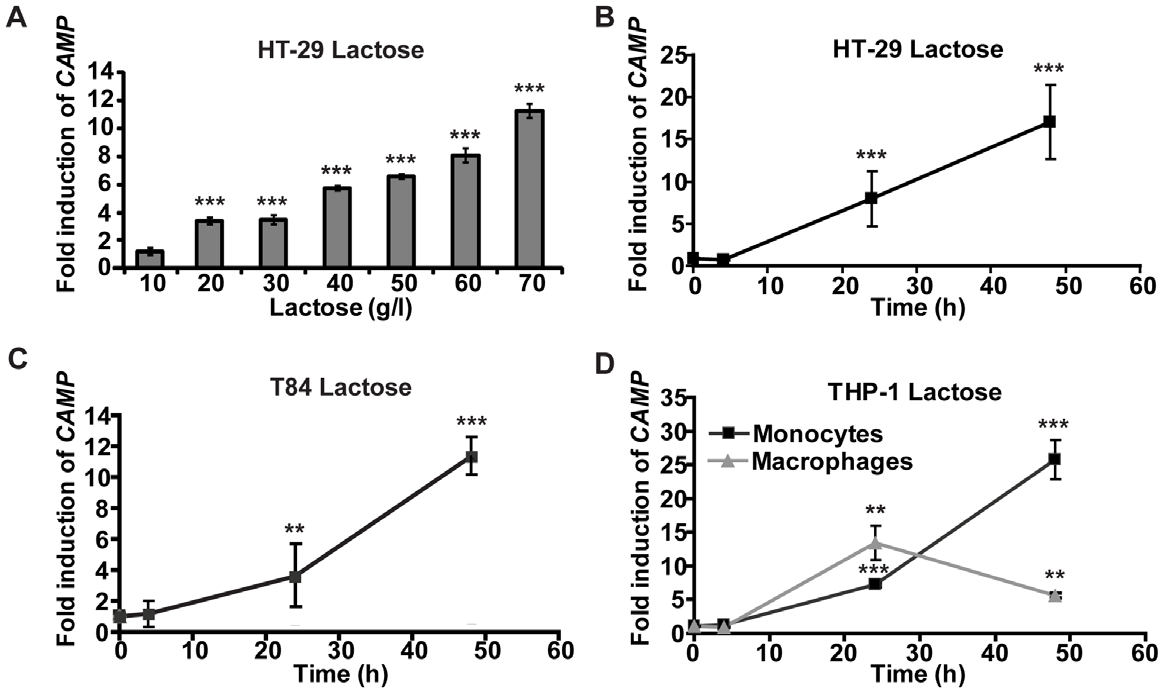
Induction of CAMP gene transcript in different cell lines stimulated with lactose. (**A**) HT-29 cells were stimulated with 10–70 g/l lactose for 48 h and *CAMP* gene transcript was monitored. At 20 g/l a 3.6-fold expression was observed that increased dose-dependently up to 11.3-fold at 70 g/l. (**B**) HT-29 cells were stimulated with 60 g/l lactose for 4, 24 and 48 h. After 24 h and 48 h an 8- and 17-fold induction of *CAMP* gene transcript was observed. No induction of *CAMP* gene was observed at 4 h. (**C**) T84 cells were stimulated with 60 g/l lactose for 4, 24 and 48 h. A 3.5-fold enhanced level of *CAMP* gene transcript was observed after 24 h and was 11.3-fold after 48 h. (**D**) THP-1 monocytes (black line) and differentiated macrophage-like THP-1 cells (grey line) were stimulated with 60 g/l lactose for 4, 24 and 48 h. In monocytes, 7.2 and 25.7-fold induction of *CAMP* gene transcript was detected after 24 h and 48 h, respectively. The macrophage-like cells exhibited a 13.5-fold induction of *CAMP* gene transcript after 24 h that declined to 5.6-fold after 48 h. (**A–D**) Displays the mean and SD of five independent experiments in duplicate.

To confirm the MS and NMR results commercially available lactose was utilized in stimulation of HT-29 cells for 48 h. At a concentration of 20 g/l a 3.6-fold significant induction (***p≤0.001) of *CAMP* gene mRNA was observed and the expression increased in a dose-dependent manner up to 11.3-fold at 70 g/l (Fig. 6A). Similarly, we investigated the time-dependent induction of *CAMP* gene transcript by 60 g/l lactose in HT-29 and T84 cells. No induction was observed at 4 h. However, after 24 h *CAMP* gene was significantly induced 8- and 3.5-fold (*** p≤0.001 and ** p≤0.005), respectively and after 48 h 17- (*** p≤0.001) and 11.3-fold (*** p≤0.001), respectively (Fig. 6B and C). Similarly, *CAMP* gene mRNA was induced in monocytic THP-1 cells and THP-1 cells differentiated into macrophage-like cells after stimulation with 60 g/l lactose. At 24 h a significant 7.2-fold induction (*** p≤0.001) in monocyte-like cells and 13.5-fold (** p≤0.005) in macrophage-like cells was observed and after 48 h 25.7- and 5.6-fold (*** p≤0.001 and ** p≤0.005), respectively (Fig. 6D). These results indicate a time- and dose-dependent induction of the *CAMP* gene with lactose. Furthermore, HT-29 and THP-1 monocytes/macrophages responded with similar kinetics of *CAMP* gene induction after stimulation with either lactose or with the hydrophilic fraction of breast milk (Fig. 1A, 2C, 6B and D). However, the induction in T84 cells was more prominent in stimulations with lactose than with the hydrophilic fraction (Fig. 2B and 6C).

### Synergism between Lactose and Sodium Butyrate or Phenylbutyrate

**Figure 7:**
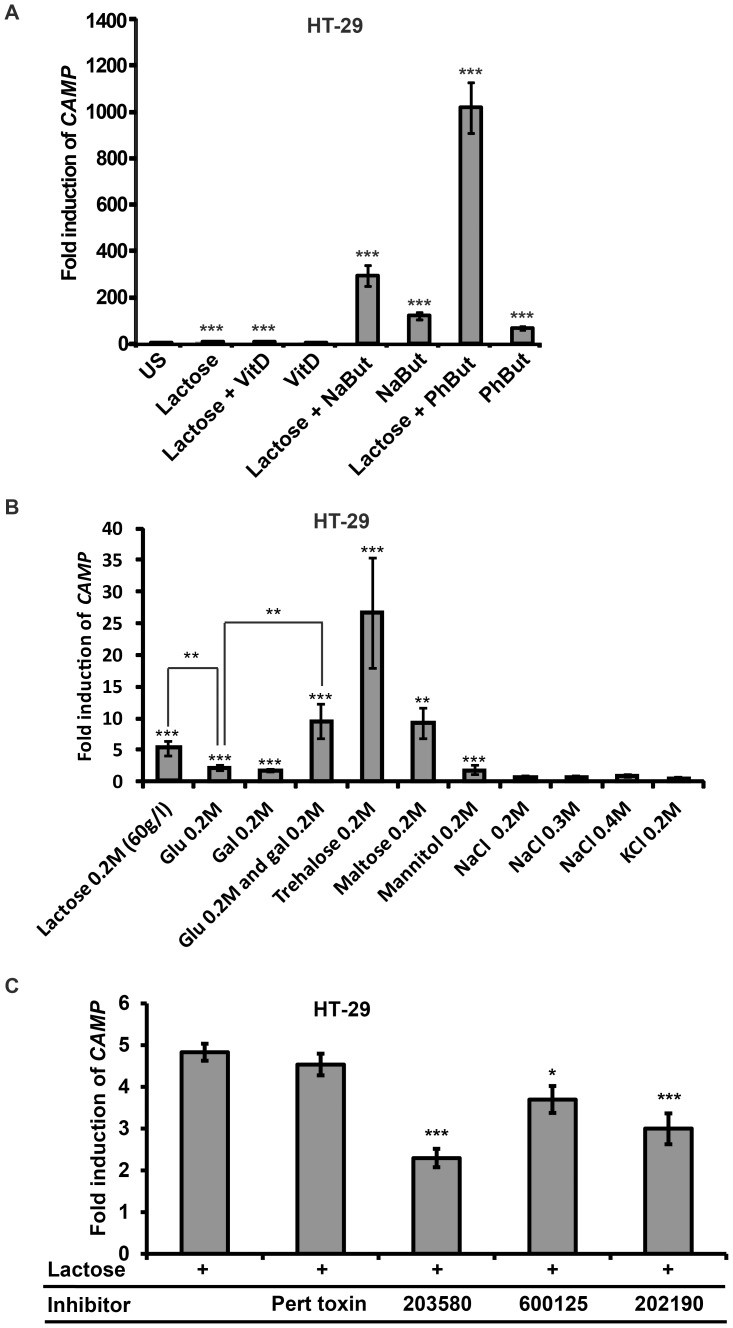
Mechanistic studies of the induction of the CAMP gene. (**A**). HT-29 cells were stimulated with 60 g/l lactose either alone or in combination with 100 nM 1,25-dihydroxyvitamin D_3_ (vitD), 2 mM phenylbutyrate (PhBut) or 2 mM sodium butyrate (NaBut) for 48 h. Lactose enhanced the level of *CAMP* gene transcript 10-fold, sodium butyrate 120-fold, whereas a combination of lactose and sodium butyrate resulted in a 294-fold *CAMP* gene induction. Stimulation with phenylbutyrate resulted in a 67-fold induction, whereas a 1018-fold induction was observed with lactose and phenylbutyrate in combination. (**B**) HT-29 cells were stimulated for 48 h with 0.2M lactose, maltose, trehalose, mannitol, glucose (Glu) and galactose (Gal). Lactose resulted in an induction of *CAMP* gene transcript 5.1-fold, trehalose 26.6-fold, maltose 9.2-fold, glucose 2.2-fold galactose 1.8-fold and mannitol 1.8 fold. Stimulation with 0.2M glucose and 0.2M galactose in combination induced *CAMP* gene transcript by 9.5-fold. No induction was observed when stimulation for 48 h with 0.2–0.4 M NaCl or 0.2 M KCl. (**C**) HT-29 cells were stimulated with 60 g/l of lactose alone or together with the following antagonists: Pertussis toxin (G-protein coupled receptors), SB203580 or SB202190 (p38 MAPK) and SP600125 (c-Jun N-terminal kinase JNK). A significant reduction in *CAMP* gene transcript was observed with both p38 inhibitors and by the JNK inhibitor SP600125. (**A and C**) Displays the mean and SD of four independent experiments in duplicate. (**B**) Displays the mean and SD of three independent experiments in triplicate.

We have previously demonstrated synergistic effects on *CAMP* gene induction when stimulating cells with vitamin D or sodium butyrate/phenylbutyrate [29], [30]. To investigate if 1,25-dihydroxyvitamin D_3_, sodium butyrate and phenylbutyrate exhibited any synergistic effects together with lactose on *CAMP* gene expression in HT-29 cells we stimulated cells with 60 g/l lactose either separately or in combination with 100 nM 1,25-dihydroxy-vitamin D_3_, 2 mM phenylbutyrate or 2 mM sodium butyrate for 48 h (Fig. 7A). Stimulation with lactose or sodium butyrate alone resulted in a 10-and 120-fold induction in *CAMP* gene expression, respectively. Interestingly, upon stimulation of cells with lactose and sodium butyrate in combination a 294-fold induction was observed. Furthermore, a 1018-fold induction of *CAMP* gene transcript was noted in cells with lactose and phenylbutyrate in combination, compared to a 67-fold induction with phenylbutyrate alone. However, no synergistic effect was observed when the cells were stimulated with 1,25-dihydroxyvitamin D_3_ in combination with lactose. These results demonstrate that there is a synergistic effect of both sodium butyrate or phenylbutyrate together with lactose in colonic epithelial cells.

### Induction of CAMP Gene Transcript by Mono- and Disaccharides

Additional mono- and disaccharides were assayed for *CAMP* gene inducing effects in HT-29 cells after 48 h stimulation (Fig. 7B). Maltose and trehalose at 0.2M were capable of inducing *CAMP* gene transcript by 9.2- and 26.6-fold, respectively, compared to a 5.1-fold induction achieved by 0.2M lactose alone. The moieties of lactose, glucose and galactose, separately revealed a minor enhancement of *CAMP* gene expression. However, 9.5-fold induction of *CAMP* gene mRNA was observed when stimulating cells with 0.2M of glucose and 0.2M galactose in combination. These results imply that the *CAMP* gene inducing effect is not restricted to lactose, but can be extended to additional carbohydrates. In order to evaluate if the lactose-mediated induction of *CAMP* was dependent on an increase in the osmolarity, HT-29 cells were stimulated with NaCl, KCl and the non-metabolizable sugar alcohol mannitol for 48 h at equimolar concentrations (or higher) as lactose (Fig. 7B). Neither NaCl, nor KCl could induce the expression of *CAMP* at any of the concentrations assayed. However, stimulation with 0.2M mannitol resulted in a significant 1.8-fold increase in the expression of *CAMP*.

### The p38 Pathway is Involved in the Lactose-mediated Induction of CAMP Gene Expression

To elucidate the signaling pathway involved in lactose-mediated *CAMP* gene induction pharmacological antagonists were utilized. HT-29 cells were stimulated with either 60 g/l lactose alone or together with pertussis toxin, an antagonist to G-protein coupled receptors, or with antagonists to p38 MAPK (SB203580 or SB202190) or SP600125, an antagonist to c-Jun N-terminal kinase (JNK). A significant suppression in lactose-mediated *CAMP* gene expression was observed with both p38 antagonists (*** p≤0.001) and with the JNK antagonist SP600125 (* p≤0.05) (Fig. 7C), indicating that lactose-mediated induction of *CAMP* gene is dependent on p38 and to some extent on JNK pathways.

## Discussion

In this study human breast milk was found to induce the expression of the *CAMP* gene encoding LL-37 in colonic epithelial cell lines and THP-1 monocytes/macrophages. After isolation lactose was identified by mass spectrometry and NMR as the inducing component. The *CAMP* gene inducing capacity of commercially available lactose was comparable to that of breast milk and dependent on p38- and JNK- pathways. Interestingly, the composition of milk changes with days postpartum: the concentration of lactose increases considerably from colostrum (∼40 g/l) to transitional milk (∼65 g/l) and is further increased in mature milk (∼75 g/l) [31]. This correlates with the increased *CAMP* gene expression observed with the hydrophilic fraction of mature milk (Fig. 3A and B), further supporting that lactose in breast milk is a major inducer of the *CAMP* gene. Albeit, there are most likely additional AMP-inducing compounds in breast milk. This may explain the residual *CAMP* gene inducing activity observed in material from the chromatographic fractions (Fig. 4B and C).

It is well known that lactose is first hydrolyzed into its glucose and galactose moieties by lactase, located in the mucosa of the small intestine [32]. Our results demonstrate no detectable levels of lactose in fecal samples of both one and four week old infants (Table 1). This suggest an efficient hydrolysis of lactose in the small bowel, which is in agreement with previous reports [33]. Hoerver, we cannot rule out the possibility that a part of the lactose is internalized by the colonic epithelial cells.

**Table 1:** Galactose and lactose levels in feces collected from neonates 1 or 4 weeks after birth. Displays the mean of samples analyzed in triplicate**.**

Feces sample	Collecting at age	Galactose	Lactose
(number)	(weeks)	(g/l)	(g/l)
1	1	2.4	Not detected
2	1	6.2	Not detected
3	1	5.9	Not detected
4	1	6.5	Not detected
5	1	3.7	Not detected
6	4	3.5	Not detected
7	4	6.3	Not detected
8	4	5	Not detected
9	4	5.5	Not detected
10	4	4.3	Not detected

In addition to lactose, the levels of galactose were determined in the same feces samples and were found to be 1–7 g/l (Table 1), revealing an incomplete absorption of this monosaccharide in the gut. Interestingly, we observed that an equimolar concentration of a combination of glucose and galactose induced *CAMP* gene expression to the same extent as lactose. Furthermore, our results indicate that also maltose induced *CAMP* gene transcript to the same extent as lactose and that trehalose can even surpass lactose in *CAMP* gene-inducing capacity on an equimolar basis.

Human breast milk provides the infant with nutrients, protective components and immune effectors, defending the infant from infections and promotes the establishment of an optimal normal flora [34], [35]. Here we demonstrated a potential additional function of breast milk carbohydrates in inducing the gene expression of *CAMP* and its gene product hCAP-18 (the precursor of the potent AMP LL-37). We also demonstrated that the inducing effect of lactose (Fig. 7A) and galactose (data not shown) was enhanced in the presence of butyrate, an established *CAMP* gene inducer and a product of colonic bacterial fermentation of dietary fibers and lactose.

AMPs, including LL-37, have been shown to be crucial components in gut homeostasis by eliminating pathogens. In addition, AMPs, e.g., human defensins 5, have been shown to determine the composition of the intestinal microbiota [36]–[39], suggesting that also LL-37 may have this effect. In this study, only the proform hCAP-18 was detected in the supernatants of HT-29 cells stimulated with breast milk (Fig. 1C). However, we have previously demonstrated that the active LL-37 peptide is present in neonatal feces, suggesting a release of this AMP in the neonatal gut *in vivo* [6]. The expression of AMPs in epithelial cells is regulated by nutrients and microbes, emphasizing the importance of environmental factors in early human intestinal homeostasis. Actually, the onset of AMP expression in the neonatal gut is enhanced in the infant intestine shortly after birth [6], [40]. We hypothesize that lactose- and galactose-mediated induction of the *CAMP* gene is important for establishing surface immune defense in the newborn. Interestingly, human milk oligosaccharides (HMOs) have been suggested to harbor prebiotic effects by promoting the establishment of an optimal normal flora [41]. HMOs have also been reported as blocking microbial anchoring to host epithelia, but can also affect expression of glycan-induced genes [42]. This last function is in line with our results, establishing milk carbohydrates as modulators of AMP expression at the host-microbe interface.

When we investigated the signaling pathway of lactose-mediated *CAMP* gene induction (Fig. 7C) we observed that pertussis toxin did not inhibit the upregulation, indicating that the lactose mediated induction is not dependent on G-protein coupled receptors [43]. The inhibition experiments further suggest that mainly p38, and to some extent JNK pathway, are involved. Known inducers of the p38 and JNK pathways are proinflammatory cytokines, lipopolysaccharide and osmotic shock [44]. Since osmotic shock may be involved in the pathology of lactose intolerance, it was of relevance to investigate such an effect [45].Thus, we stimulated HT-29 cells with equimolar concentrations of either glucose or galactose, resulting in significantly lower induction of *CAMP* gene expression compared to lactose. We also stimulated these cells with equimolar (or higher) concentrations of additional agents that increase the osmolarity, *i.e.* NaCl, KCl (Fig. 7B) without any induction of *CAMP* gene expression. However, as opposed to the impermeant *CAMP*-inducing carbohydrates (trehalose and mannitol), these salts are allowed to enter the cell and may thus stress intracellular systems that are not affected by an impermeant carbohydrate. This makes it difficult to exclude an osmosis-dependent mechanism. Albeit, the differential inducing effects of equimolar concentrations of the impermeant *CAMP*-inducing carbohydrates suggest that additional mechanisms are involved, such as receptor-mediated activation. Furthermore, trehalose has been shown to promote mucin-producing, goblet-like maturation of HT-29 cells that might be involved in the mechanism of carbohydrate-mediated *CAMP*-induction [46].

When testing infant formulas of different brands, we found the median level of *CAMP* gene expression was only half of that for mature breast milk (2.2- vs. 3.9-fold) (Fig. 3A). However, no correlation between formula lactose content and *CAMP* gene induction was observed. An explanation for this might be that infant formulas often contain additives that in themselves can modulate the neonatal immune system. Examples of such milk formula additives are long-chain polyunsaturated fatty acids [47], nucleotides [48] and probiotics [49].

Primary lactose intolerance is an inherited trait present in most of the world’s population and is characterized by a gradual decline in lactase level during maturation [32]. However, small bowel injury, such as celiac disease, inflammatory bowel disease (IBD) or gastroenteritis can lead to secondary lactose intolerance [50] with reduced lactase activity. Lactose intolerance will lead to an increased concentration of lactose in the colon, resulting in symptoms such as bloating, flatulence and diarrhea. We propose that some of the observed symptoms may in fact be an effect of perturbation of the microflora in response to increased expression of *CAMP* in the colon mediated by lactose. This effect may be further exacerbated by butyrate, an established *CAMP* gene inducer and a product of colonic fermentation of dietary fibers and lactose [51], [52]. It has been established that IBD can be exacerbated by a lactose-rich diet [53]. Our results may be a part of an explanation of these symptoms, since we here demonstrate that lactose is able to induce AMP expression, which has been linked to IBD [54]. In addition, the peptides *per se* have in some settings been shown to be pro-inflammatory [55], [56]. The ingestion of trehalose may result in diarrhea in patients diagnosed with trehalase deficiency [57]. This is consistent with the hypothesis that undigested trehalose may disturb the microbial homeostasis of the intestine through increased levels of AMP expression.

One further implication of our study is related to pharmaceutical products that frequently include lactose as an excipient or in placebo. The presence of lactose in medications might affect the results of the treatments and studies due to the biological effects of lactose that we have here demonstrated.

We conclude that lactose in human milk stimulates expression of *CAMP* and its gene product hCAP-18 in cell culture, supporting the hypothesis that this dominant milk carbohydrate may play a role in the developing intestinal microbiota and innate immune response of the neonate.
